# p19-Targeting ILP Protein Blockers of IL-23/Th-17 Pro-Inflammatory Axis Displayed on Engineered Bacteria of Food Origin

**DOI:** 10.3390/ijms19071933

**Published:** 2018-07-01

**Authors:** Katja Škrlec, Petra Zadravec, Marie Hlavničková, Milan Kuchař, Lucie Vaňková, Hana Petroková, Lucie Křížová, Jiří Černý, Aleš Berlec, Petr Malý

**Affiliations:** 1 Department of Biotechnology, Jožef Stefan Institute, Jamova 39, SI-1000 Ljubljana, Slovenia; katja.skrlec@ijs.si (K.Š.); petrazadravec@gmail.com (P.Z.); ales.berlec@ijs.si (A.B.); 2 Graduate School of Biomedicine, Faculty of Medicine, University of Ljubljana, SI-1000 Ljubljana, Slovenia; 3 Laboratory of Ligand Engineering, Institute of Biotechnology of the Czech Academy of Sciences, v. v. i., BIOCEV Research Center, Průmyslová 595, 252 50 Vestec, Czech Republic; marie.hlavnickova@ibt.cas.cz (M.H.); milan.kuchar@ibt.cas.cz (M.K.); lucie.vankova@ibt.cas.cz (L.V.); hana.petrokova@ibt.cas.cz (H.P.); lucie.krizova@ibt.cas.cz (L.K.); 4 Laboratory of Structural Bioinformatics of Proteins, Institute of Biotechnology of the Czech Academy of Sciences, v. v. i., BIOCEV Research Center, Průmyslová 595, 252 50 Vestec, Czech Republic; jiri.cerny@ibt.cas.cz; 5 Faculty of Pharmacy, University of Ljubljana, Aškerčeva 7, SI-1000 Ljubljana, Slovenia

**Keywords:** lactococcus, binding protein, albumin-binding domain, cytokine, IL-23, surface display

## Abstract

IL-23-mediated Th-17 cell activation and stimulation of IL-17-driven pro-inflammatory axis has been associated with autoimmunity disorders such as Inflammatory Bowel Disease (IBD) or Crohn’s Disease (CD). Recently we developed a unique class of IL-23-specific protein blockers, called ILP binding proteins that inhibit binding of IL-23 to its cognate cell-surface receptor (IL-23R) and exhibit immunosuppressive effect on human primary blood leukocytes ex vivo. In this study, we aimed to generate a recombinant *Lactococcus lactis* strain which could serve as in vivo producer/secretor of IL-23 protein blockers into the gut. To achieve this goal, we introduced ILP030, ILP317 and ILP323 cDNA sequences into expression plasmid vector containing USP45 secretion signal, FLAG sequence consensus and LysM-containing cA surface anchor (AcmA) ensuring cell-surface peptidoglycan anchoring. We demonstrate that all ILP variants are expressed in *L. lactis* cells, efficiently transported and secreted from the cell and displayed on the bacterial surface. The binding function of AcmA-immobilized ILP proteins is documented by interaction with a recombinant p19 protein, alpha subunit of human IL-23, which was assembled in the form of a fusion with Thioredoxin A. ILP317 variant exhibits the best binding to the human IL-23 cytokine, as demonstrated for particular *L.lactis*-ILP recombinant variants by Enzyme-Linked ImmunoSorbent Assay (ELISA). We conclude that novel recombinant ILP-secreting *L. lactis* strains were developed that might be useful for further in vivo studies of IL-23-mediated inflammation on animal model of experimentally-induced colitis.

## 1. Introduction

IL-23/Th17 pro-inflammatory axis plays a central role in pathogenesis of several autoimmune diseases such as psoriasis, psoriatic arthritis and rheumatoid arthritis [1,2] but it is also closely related to development of inflammatory bowel disease and Crohn’s disease [3,4,5]. Human IL-23 cytokine, heterodimer of p19 and p40 proteins, stimulates differentiation of naive Th cells into Th17 cell population by activation of IL-23 receptor-mediated signaling, leading to secretion of a cocktail of inflammatory mediators including IL-17, IL-22 and chemokines [6,7]. Therefore, blocking of IL-23 signaling represents a crucial step in therapeutic intervention and treatment of autoimmune diseases. Recently it has been demonstrated that neutralizing antibodies targeting IL-23 such as Ustekinumab (Stelara) or IL-17A such as Secukinumab (Cosentyx) exhibit a substantial effect in the treatment of severe forms of psoriasis [8,9]. 

Alternatively, several laboratories develop non-immunoglobulin blockers of IL-23 or IL-23R using directed evolution based on selection of high-affinity binding proteins from highly complex combinatorial libraries derived from small protein domain scaffolds. Among them, anti-IL-23 Nanobody demonstrate anti-inflammatory effect in mouse model of contact hypersensitivity [10], anti-IL-23 Adnectin exhibit high-affinity IL-23 binding [11], anti-IL-23 Alphabody binder prevents topical inflammation in mice [12] and albumin-binding domain (ABD)-derived antagonists of IL-23 receptor (REX ligands) suppress IL-23-stimulated expansion of primary human peripheral blood mononuclear cells in vitro [13]. Recently we developed a novel collection of ABD-derived proteins called ILP binders targeting alpha subunit of IL-23, p19 protein. These proteins were shown to suppress production of primary human Th17 cells ex vivo upon stimulation with IL-23 and IL-2 [14]. 

Local delivery and neutralization of pro-inflammatory mediators in situ, in gastrointestinal tract, was shown to be beneficial in animal models of inflammatory bowel disease [15]. Oral administration of protein therapeutics is not feasible due to extensive protein degradation in stomach and duodenum, high dosage requirements and high price. Advanced oral protein delivery systems are therefore required [16]. Better protein stability and cost-efficiency could be achieved by using recombinant bacteria that would serve as protein producers and delivery systems, at the same time. Administration of live bacteria is associated with concerns for safety; the latter should therefore be, beside effective protein delivery, of prime importance in selection of appropriate bacterial species [17]. The bacteria should have no or minimal pathogenic potential, should produce no toxins or immune response triggers (such as lipopolysaccharide), and should preferably be of food origin.

Lactic acid bacteria (LAB) have a long history of safe usage as part of human food. LAB are Gram-positive bacteria from genera *Lactococcus*, *Lactobacillus*, *Leuconostoc*, *Pediococcus* and *Streptococcus* that produce lactic acid as an end product of carbohydrate metabolism [18]. Several of them are part of human intestinal and vaginal microbiota. Some LAB are used as probiotics that are defined as “live microorganisms that when administered in adequate quantity confer health benefit on host” [19].

*Lactococcus lactis* is a model lactic acid bacterium with well-developed tools for genetic engineering. It has been widely used in the food industry as a starter for the production of cheese. Apart from its industrial importance [20], *L. lactis* has also been recognized in recent years as a potential probiotic with beneficial effects in experimental colitis [21,22], which supports its role as a delivery vehicle in IBD treatment. *L. lactis* can survive passage through the intestinal tract but does not colonize it [23]. Intrinsic probiotic efficacy of *L. lactis* can be further strengthened by genetic engineering, and several proof-of-principle applications have already been developed [24,25,26]. Delivery of anti-TNFα Nanobody [27], anti-TNFα Affibody [28], trefoil factors [29] and elafin [30], by recombinant *L. lactis*, have demonstrated effectiveness in DSS-induced colitis animal model of IBD. Delivery of anti-inflammatory cytokine IL-10 has been tested in human clinical trial [17,31].

Therapeutic proteins can be either secreted [27,31] or displayed on the bacterial surface [32,33]. Display of binders enables removal of the targeted mediators, together with bacteria, following bacterial passage through the intestine and their excretion. Surface display can be achieved by different molecular means [34]. A well-established approach consists of fusing the protein binder with Usp45 secretion signal [35] and C-terminal part of lactococcal AcmA protein containing 3 LysM repeats that serve as surface anchor [36,37]. In our recent work we verified the function of IL-23-binding Adnectin on lactic acid bacteria [38] and we extended this study in the current work by displaying three-helix bundle ABD scaffold-derived IL-23 binders.

## 2. Results

### 2.1. Molecular Assembly of Recombinant p19 Protein

**Figure 1:**
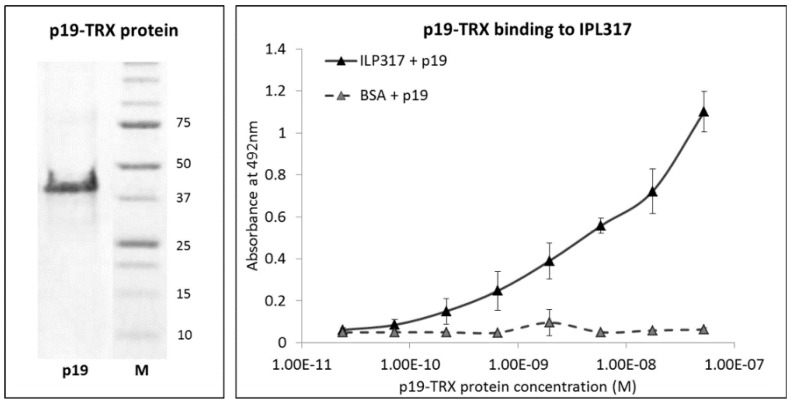
Binding of p19-TRX fusion protein to immobilized ILP317 variant in ELISA. Left: Recombinant p19 protein, alpha subunit of human IL-23, was produced as a fusion protein with Thioredoxin A. Protein was expressed in *E. coli* BL21(λDE3), purified from inclusion bodies and refolded from 8 M urea extracts. Final product of calculated molecular weight 40 kDa is shown as a stained band after SDS polyacrylamide gel electrophoresis. Right: 96-well Polysorp ELISA plate was coated with the ILP317 protein variant in the form of a fusion with TolA-Avitag protein. p19-TRX was used as an analyte, detected by anti-IL-23 (p19) polyclonal antibody and anti-mouse IgG-HRP conjugate. The result represents three individual measurements and the error bars indicate standard deviations.

Previous studies demonstrated that p19 protein, alpha subunit of IL-23, can be produced as a recombinant protein produced in bacterial host cells and that it retains folding recognized by IL-23 receptor on THP-1 cells or by IL-23R-IgG chimera in Enzyme-Linked ImmunoSorbent Assay (ELISA). Therefore, as a molecular probe for ILP protein binder detection, we generated a p19 fusion protein using bacterial plasmid carrying sequences coding for human p19 protein (alpha subunit of IL-23), assembled in the form of a fusion with Thioredoxin A (TRX). For the construction of this protein, we used the same plasmid vector as it was used before for the generation of p19 fusion protein with maltose-binding protein (p19-MBP) [14]. The corresponding purified p19-TRX protein, produced with a double His6-tag at the *N*-terminus, was refolded from urea extracts and tested in ELISA for specific recognition of ILP proteins (Figure 1). This 40 kDa protein was then used as a detection tool for the surface display of ILP binders on *L. lactis*.

### 2.2. Expression of ILP-Fusion Proteins in L. lactis

**Figure 2:**
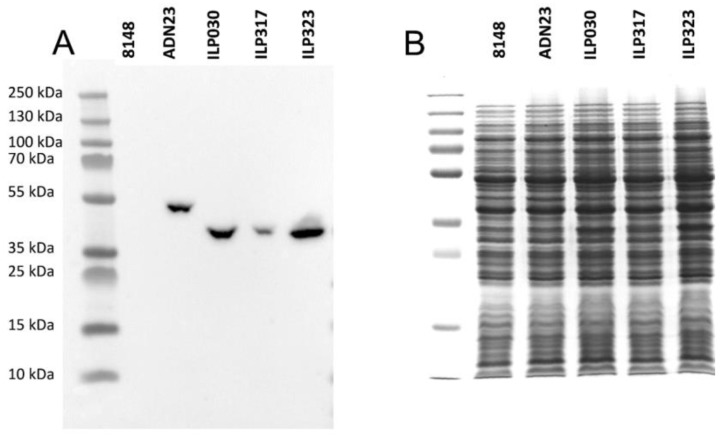
Analysis of protein expression by Western blot (**A**) and Coomassie Brilliant Blue-stained SDS PAGE gel (**B**) of lysates of *L. lactis* cells expressing ILP binding proteins and ADN23. All proteins are in fusion with Usp45 secretion signal, FLAG tag and LysM-containing cA surface anchor.

To verify whether IL-23-specific ILP protein binders can be produced in *L. lactis* host cells, DNA sequences coding for ILP proteins (ILP030, ILP317 and ILP323 variants) were genetically fused to Usp45 secretion signal and C-terminal domain of AcmA protein (cA) containing LysM repeats (LysM) for peptidoglycan anchoring. Fusion genes were under the control of inducible nisin promoter. To simplify detection of particular ILP proteins, FLAG-tag sequence consensus was added between secretion signal and ILP coding sequences. Previously reported construct for the display of IL-23-binding Adnectin variant ADN23 [38] was also modified by inserting FLAG-tag. All three ILP fusion proteins, as well as the Adnectin fusion protein, were detected in bacterial cell lysates after nisin-stimulated induction using anti-FLAG antibodies (Figure 2A). No signal could be detected in empty plasmid pNZ8148-containing control cells. Also, the fusion proteins could not be observed in Coomassie Brilliant Blue stained gel (Figure 2B). However, visualization of protein lysates demonstrates their uniform concentration. The acquired data documents that all ILP proteins are expressed. Among them, ILP317-containing fusion protein was expressed at the lowest level.

### 2.3. Display of ILP-Fusion Proteins on the Surface of L. lactis

**Figure 3:**
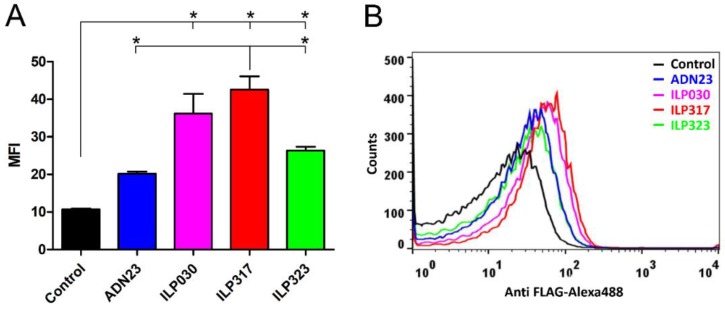
Flow cytometry of *L. lactis* cells displaying ILP proteins, or ADN23, detected with Anti-FLAG tag antibodies demonstrating mean fluorescence intensity (MFI; **A**) or a shift in fluorescence intensity (**B**). Vertical bars denote standard error. Significant differences (*p* < 0.05) are marked with an asterisk.

To investigate a secretion efficacy of the particular ILP variants produced in lactococcal cells, cell-surface display of AcmA-anchored ILP proteins and ADN23 was used. The bound proteins were detected with anti-FLAG antibodies using flow cytometry. The data confirm that all ILP proteins as well as ADN23 Adnectin were effectively secreted and displayed in comparison to the used negative control (Figure 3A,B). However, they were displayed in different amounts. The results document that all three ILP binding proteins were displayed in higher amounts than ADN23. The best cell-surface display was achieved with ILP317 variant, followed by ILP030 and ILP323.

### 2.4. Binding of Recombinant p19 by ILP-Displaying L. lactis

**Figure 4:**
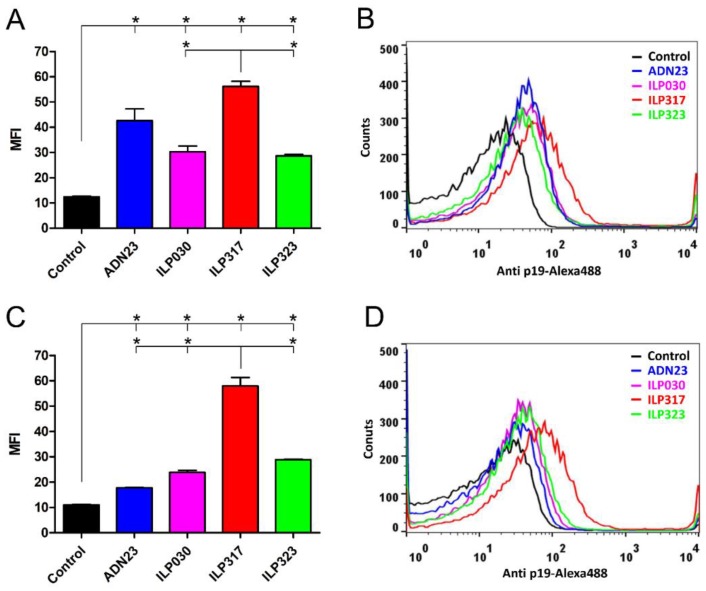
Flow cytometry of *L. lactis* cells displaying ILP proteins, or ADN23, without FLAG tag (**A**,**B**) or with FLAG tag (**C**,**D**) detected with recombinant p19-TRX protein. Mean fluorescence intensity (MFI; **A**,**C**) or a shift in fluorescence intensity (**B**,**D**) are depicted. Vertical bars denote standard error. Significant differences (*p* < 0.05) are marked with an asterisk.

The ability of ILP-displaying *L. lactis* to bind the p19 protein was tested by flow cytometry. To achieve this goal, a recombinant p19-TRX fusion protein was used as a useful molecular probe. The bound p19 was detected by a specific anti-human IL-23 p19 antibody. The highest binding of p19 was observed with ILP317, followed by ADN23 (without FLAG tag). Lower binding was demonstrated with ILP030 and ILP323 (Figure 4A, B). The presence of FLAG tag had a little effect on the ability of ILP proteins to bind p19. However, the binding of p19 was severely hindered when FLAG tag was attached to the ADN23 (Figure 4C, D).

### 2.5. Removal of IL-23 by ILP-Displaying L. lactis

**Figure 5:**
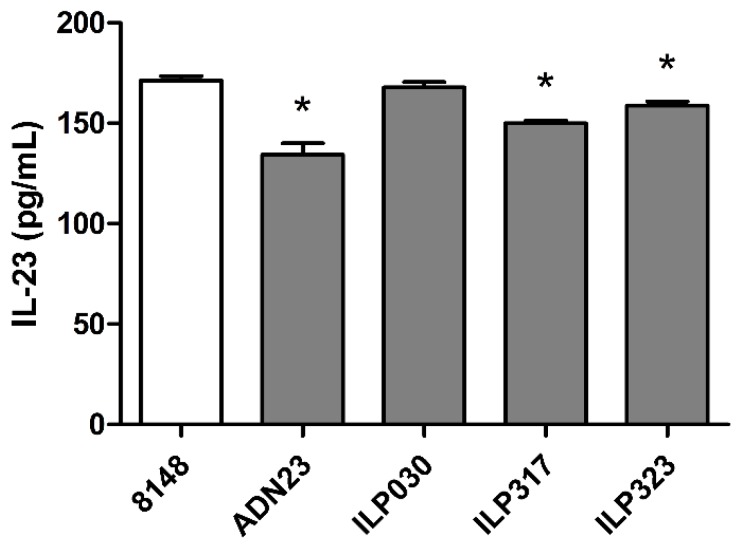
ELISA-determined removal of IL-23 from the solution by *L. lactis* displaying ILP binding proteins or ADN23. Concentration of remaining IL-23 is shown. Vertical bars denote standard error. Significant difference (*p* < 0.05) in comparison to control (8148) is marked with an asterisk.

To verify that ILP variants attached to *L. lactis* bacteria recognize not only recombinant p19-TRX fusion protein, but also secreted IL-23 cytokine, we performed cell-surface binding test. The binding and removal of IL-23 from the solution by 1 × 10^10^ ILP-displaying *L. lactis* was evaluated by ELISA and compared to ADN23-displaying *L. lactis*. The removal of IL-23 was the most effective with ADN23-displaying bacteria (Figure 5). Removal of IL-23 was more effective with ILP317-displaying bacteria than with ILP323-displaying bacteria. No significant removal of IL-23 could be observed with ILP030-displaying bacteria. 

## 3. Discussion

In the present work, we have developed a set of new recombinant bacteria of food origin with the ability to bind p19 protein, an alpha-subunit of the pro-inflammatory cytokine IL-23. IL-23/Th17 pro-inflammatory axis plays an important role in the pathogenesis of inflammatory bowel disease. It has been demonstrated that the mucosal delivery of anti-inflammatory cytokines or engineered protein blockers of pro-inflammatory cytokines can effectively decrease inflammation in the mouse models of disease, and that mucosal delivery can be achieved by recombinant bacteria, preferably of confirmed safety and of food origin. We have previously used lactic acid bacterium *Lactococcus lactis* to deliver binders of TNFα, IL-17A, IL-23 and chemokines [32,33,38]. In the present work we displayed our recently developed ILP binders of p19 subunit of IL-23 [14] on the surface of *L. lactis* and confirmed their ability to bind the p19 subunit that was isolated from *E. coli* in the form of thioredoxin fusion, as well as their ability to bind and remove IL-23 in vitro.

ILP binders were originally selected from a highly complex combinatorial library derived from albumin-binding domain (ABD) scaffold using ribosome display where the target p19 protein was produced as a bacterial fusion product of maltose-binding protein (p19-MBP) [14]. However, this soluble protein is not suitable for cell-surface competition binding assays performed with IL-23 receptor-expressing human and mouse cell lines as MBP interacts with glycans of plasma membrane receptors. As double-His6-p19 protein expression is rather poor and this protein is not sufficiently stable, we produced another type of p19 fusion protein using thioredoxin A. This p19-TRX was well produced in *E. coli* as insoluble protein and after refolding, it was recognized by anti-p19 antibody in ELISA. Stability of this product was increased further by a carrier protein added into solutions. We used, therefore, p19-TRX protein as a molecular probe for detection of ILP variants presented on the surface of *L. lactis*.

Previously developed IL-23-binding *L. lactis*, displaying Adnectin against IL-23 (ADN23) [38] was complemented with the display of three additional binders, directed against p19 subunit of IL-23, ILP030, ILP317 and ILP323. All three ILP binders were effectively displayed on the surface *of L. lactis* in the form of FLAG-tag fusion. The amount of surface display of ILP binders was higher than that of ADN23. All three ILP binders, as well as ADN23, were able to bind recombinant p19 subunit of IL-23. However, the binding depended on the presence of FLAG tag. After its removal, the binding ability of displayed ADN23 increased, probably due to the elimination of steric hindrances, as it is known that the N-terminus of ADN23 is important in the interaction with p19 [11]. Lower binding of p19 by ADN23 can also be explained by the fact that ADN23 interacts with both subunits of IL-23, p19 and p40 [11]. 

ILP317 emerged as the most effective binder of p19 subunit in the *L. lactis* host system. Although its expression level was lower than that of ILP030 or ILP323, it demonstrated the highest level of surface display (established by anti-FLAG antibody), as well as the highest ability to bind recombinant p19 subunit among the tested binders. The FLAG tag had a little effect on binding of p19 with ILP317-displaying lactococci. ILP317 was also the most effective ILP binder of IL-23, when displayed on the surface of lactococci. Its binding efficacy was compared to that of ADN23 (being only slightly lower). ADN23 used in this study was identified as a high-affinity human IL-23 binder which binds to the interface between p19 and p40 subunits with 2 nM K_d_ ([11]. This binding region is, however, far from that one essential for IL-23 recognition by a distal IgG-like domain of the IL-23 receptor (based on 5MZV crystal structure by [39]) and, therefore, is not expected to suppress IL23/IL23R interaction. In our study, ADN23 was intended as a suitable IL-23-binding probe useful for *L. lactis*-secreting ILP strains characterization. On the other hand, our results suggest that ILP317 would be a suitable candidate for future testing of the in vivo mucosal neutralization of IL-23, and possible anti-inflammatory activity in an animal model of IBD. However, relatively low amount of the in vitro removed IL-23 may require further optimization of the binder affinity or of the bacterial concentration.

**Figure 6:**
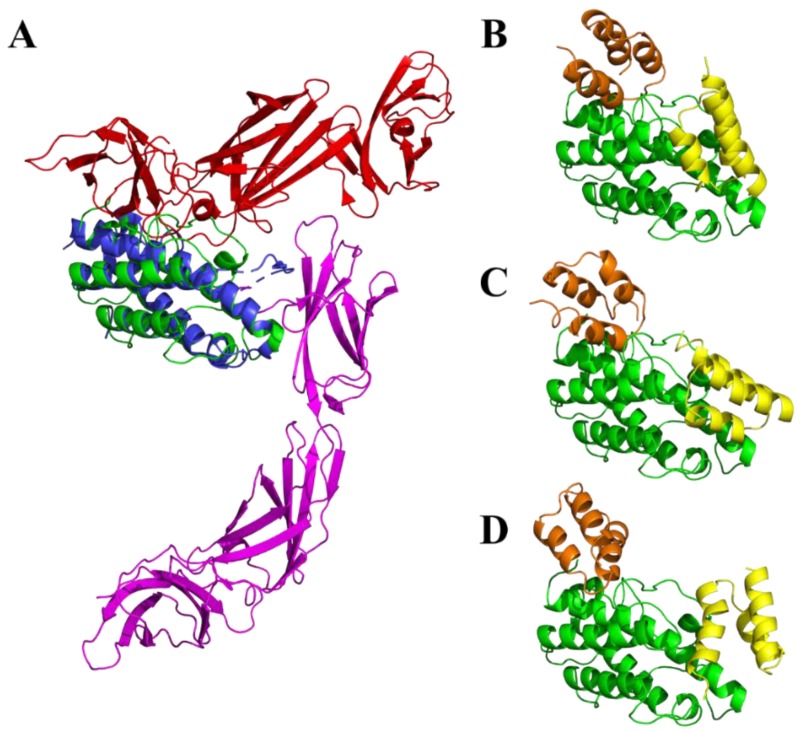
Modeling of p19/ILP interactions. (**A**) Comparison of homology model of mouse p19 protein (green) to crystal structure of human IL-23/IL-23R complex (PDB ID 5MZV [39]), with p19 (blue), p40 (red) and IL-23R (magenta). Representative binding modes from docking of ILP variants ILP030 (**B**), ILP317 (**C**) and ILP323 (**D**) to the homology model of the mouse p19 protein (green) are shown. Yellow and orange colors indicate common binding areas predicted on the mouse p19. The residues involved in the interaction with the orange binder overlap with p19/p40 interaction interface (Figure 6A blue/red) while the yellow binding mode corresponds to p19/IL-23R interface (Figure 6A blue/magenta).

ILP binding proteins were originally targeted to human p19 protein. In our previous study we identified probable binding modes for the all ILP proteins by docking to our homology model of the human IL-23/IL-23R complex [14]. We built a homology model for IL-23R using the IL-6 receptor as a template and performed docking of p19 as well as the IL-23 cytokine with IL-23R. The results of docking suggested the most probable binding site located at the extracellular Ig-like domain and distal domain of the fibronectin-type III of IL-23R. This agrees with the recently deposited crystal structure of the human IL-23/IL-23R complex (PDB ID 5MZV [39]) shown in Figure 6A. However, the structure of mouse IL-23/IL-23R complex is still not available. We, therefore, generated a homology model of the mouse p19 and relaxed its structure using short molecular dynamics simulation. To investigate the most probable binding regions for the all ILP binding proteins, we performed docking of ILP030, ILP317, and ILP323 to the mouse p19 model. The docking revealed two principal binding regions (Figure 6B–D). While the first one overlaps with p19/p40 interaction interface in the human IL-23/IL-23R complex (Figure 6A blue/red) and is, therefore, inaccessible in IL-23 due to a steric hindrance by p40, the second one corresponds to p19/IL-23R interface (Figure 6A blue/magenta) and is exposed for binding of ILP blockers.

As our intention is to verify an inhibitory potential of ILP proteins in vivo using mouse model of experimentally induced colitis, there is a question about the efficacy of ILP blockers on the mouse IL-23. Sequence comparison of human p19 (UniProt Q9NPF7) and mouse p19 (Q9EQ14) revealed overall protein sequence identity 71.9% and the similarity 90.4%. Based on our previously described p19-ILP complexes [14] we generated a homology model of mouse p19 complexes. This allowed us to identify 28 amino acid residues forming a “common interface” between p19 and particular ILP variants. In this amino acid stretch, only seven amino acids differ between human and mouse p19 proteins. However, six of them do not change the charge: 49-51PLV to 49-51APA, D55 to N55, S114 to A115, 159L to 160P, and 163F to 164S. The analysis of human p19-ILP complexes demonstrated that most of the contacts between p19 and ILP proteins are mediated by charged amino acid residues. Therefore, we do not expect major changes in the binding affinity/neutralization efficacy of the used ILP protein blockers in the mouse model. Modeling of the mouse p19 suggests that sequence differences between mouse and human p19 protein cause only minor structural differences (Figure 6A, blue/green). These results support an expectation that ILP variants secreted by modified *L. lactis* cells can serve as anti-inflammatory blockers during stimulated intestinal inflammation in mouse. However, this has yet to be verified.

## 4. Materials and Methods 

### 4.1. Bacterial Strains, Media and Growth Conditions

The bacterial strains used in this study are shown in Table 1. *Lactococcus lactis* NZ9000 was grown at 30 °C in M17 medium (Merck, Darmstadt, Germany) supplemented with 0.5% glucose (GM-17) without agitation or in the same medium solidified with 1.5% agar. Electroporation of *L. lactis* was performed according to [40], using Gene Pulser II apparatus (Bio-Rad, Hercules, CA, USA). To maintain selection pressure on transformation, 10 µg/mL chloramphenicol was added to the growth medium. *E. coli* strain DH5α was grown at 37 °C with agitation in lysogeny broth (LB) medium supplemented with 100 µg/mL ampicillin. For bacterial strains, plasmids and oligonucleotide primers used in the study see Table 1.

### 4.2. DNA Manipulation and Plasmid Construction

Restriction enzymes and T4 DNA ligase were purchased from Fermentas or New England Biolabs. PCR amplifications were performed with Taq polymerase (Fermentas, Waltham, MA, USA) or Phusion Hot Start polymerase (Thermo Fisher Scientific, Waltham, MA, USA) according to the manufacturer´s protocols. PCR products were routinely introduced into pGEM-T Easy (Promega, Madison, WI, USA) or pJET1.2 (CloneJET PCR Cloning Kit, Fermentas, Waltham, MA, USA) for sequencing and further cloning. Plasmid DNA was isolated with NucleoSpin Plasmid (Macherey-Nagel, Duren, Germany), with an additional lysozyme treatment step in the case of *L. lactis*. Nucleotide sequencing was performed by GATC. Primers (IDT) and plasmids are listed in Table 1. The *ilp030*, *ilp317* and *ilp323* genes were amplified by PCR from original plasmids carrying sequences of ILP030, ILP317 and ILP323 proteins [14] using ILP030-F/ILP030-R, ILP030-F/ILP317-R and ILP030-F/ILP323-R primer pairs, respectively. All amplicons were cloned to pSDLBA3b [32] via BamHI/EcoRI restriction sites. To insert FLAG tag nucleotide sequence between usp45 signal sequence and ilp sequences in pSD-ILP plasmids, the usp45 sequence was amplified from pSDLBA3b using primers Usp1-NcoI/FLAG_Bam_R. The amplicon was digested with NcoI/BamHI and cloned into equally treated plasmids pSD-ILP030, pSD-ILP317, pSD-ILP323 and pSD-ADN23, thereby yielding plasmids pSD-ILP030-FLAG, pSD-ILP317-FLAG, pSD-ILP323-FLAG and pSD-ADN23-FLAG, respectively. The DNA sequence encoding p19 was inserted into an assembled pET28b-derived vector, carrying sequence coding for 2xHis6-TRX-TEV-MCS-TEV-His6, using primers p19-F-NheI and p19-R-XhoI. *E. coli* TOP10 (Life Technologies, Carlsbad, CA, USA) host cells were transformed with the p19-cloned vector and plated on LB-agar supplemented with 60 μg/mL kanamycin. The resulting plasmid construct contains sequences coding for the final 360 amino acids p19-TRX fusion product as follows:

MVPHHHHHHSRAWRHPQFGGHHHHHHARHMMSDKIIHLTDDSFDTDVLKADGAILVDFWAEWCGPCKMIAPILDEIADEYQGKLTVAKLNIDQNPGTAPKYGIRGIPTLLLFKNGEVAATKVGALSKGQLKEFLDANLALQENLYFQGASRAVPGGSSPAWTQCQQLSQKLCTLAWSAHPLVGHMDLREEGDEETTNDVPHIQCGDGCDPQGLRDNSQFCLQRIHQGLIFYEKLLGSDIFTGEPSLLPDSPVGQLHASLLGLSQLLQPEGHHWETQQIPSLSPSQPWQRLLLRFKILRSLQAFVAVAARVFAHGAATLSPLEKKTCTSRASSTTTTTTEIRLLTKPERKF.

### 4.3. Production of p19-TRX Fusion Protein

p19 protein was produced from an engineered plasmid construct pET-DH-TRX-p19 in the form of a fusion protein with Thioredoxin A (TRX) and a double His6-tag at the *N*-terminus (calculated Mw 40 kDa). The p19-TRX fusion protein was produced in *E. coli* BL21(λDE3) host cells in 100 mL LB medium containing 60 μg/mL kanamycin at 37 °C and shaken at 250 RPM until the culture reached OD600 = 0.6. Subsequently, the protein production was induced with 1 mM IPTG and the culture was let to grow for 4 h at 37 °C. Cells were harvested by centrifugation (6000× *g*, 20 min), washed with TN buffer (50 mM Tris-HCl and 150 mM NaCl, pH = 8) and centrifuged again (5000× *g*, 10 min). For protein extraction and purification, cell pellets were re-suspended in 10 mL TN buffer and disrupted by MISONIX 3000 sonicator. The lysates were centrifuged at 40,000× *g* for 20 min and the insoluble p19 protein was extracted with 10 mL of 8 M urea in TN buffer. The urea extract was left shaking for half an hour at room temperature and centrifuged at 40,000× *g* for 30 min. The supernatant was applied on 1 mL Ni-NTA agarose column equilibrated with TN buffer. The washing steps were performed with 10 mL of TN buffer with 8 M urea and, subsequently, with TN buffer containing 8 M urea and 20 mM imidazole. The p19 protein was then eluted with TN buffer containing 8 M urea and 250 mM imidazole. To remove imidazole, urea and Ni-ion traces, eluted samples were dialyzed in PBS buffer or stabilized by adding (1:1) SuperBlock solution (SuperBlock^®^ Blocking Buffer, 37515, Pierce Biotechnology, Rockford, IL, USA).

### 4.4. p19 Binding Assay

p19-TRX fusion protein was dialyzed into PBS or SuperBlock solution (1:1) and its binding activity was examined by ELISA. Wells of Polysorp plate (NUNC, Denmark) were coated with 10 µg/mL ILP317-TolA-Avitag protein diluted in 100 mM bicarbonate/carbonate coating buffer, pH = 9.6, and incubated at 7 °C overnight. The following day, the plate was washed with PBS buffer containing 0.05% Tween (PBST) and blocked with 1% BSA resolved in the same solution (PBSTB). Series of diluted p19-TRX samples in PBSTB were prepared and p19 binding to the immobilized ILP317-TolA-Avitag protein was detected using mouse anti-human IL-23 polyclonal antibody (clone HLT2736, BioLegend, San Diego, CA, USA) diluted in PBSTB (1:2000) followed by goat anti-mouse IgG horseradish peroxidase (HRP) conjugate (BioLegend, San Diego, CA, USA) (1:1000). The enzymatic reaction of HRP with OPD substrate (Sigma-Aldrich, St. Louis, MO, USA) in citrate buffer (3.31% Sodium citrate tribasic dihydrate, phosphoric acid until pH = 5.0) was visualized after 5 min staining, then the reaction was stopped by 2M sulfuric acid and the absorbance was measured at 492 nm.

### 4.5. Expression of Cytokine Binding Fusion Proteins in L. lactis

Overnight cultures of *L. lactis* NZ9000 transformed with the appropriate plasmid (pSD-ILP030, pSD-ILP317, pSD-ILP323, pSD-ADN23, pSD-ILP030-FLAG, pSD-ILP317-FLAG, pSD-ILP323-FLAG, pSD-ADN23-FLAG or pNZ8148–empty plasmid control) were diluted (1:100) in 10 mL of fresh GM-17 medium and grown to optical density A600 = 0.80. Fusion protein expression was induced with 25 ng/mL nisin (Fluka Chemie AG, Buchs, Switzerland). After three hours of incubation, 1 mL of culture was stored at 4 °C for flow cytometry, and the remaining cell culture was centrifuged at 5000× *g* for 10 min. The cell pellet was resuspended in 400 µL of phosphate-buffered saline (PBS, pH 7.4) and stored at −20 °C for SDS PAGE analysis or resuspended at to optical A600 = 10.0 and stored at 4 °C for enzyme-linked immunosorbent assay (ELISA). 

### 4.6. SDS PAGE and Western Blot

SDS PAGE was performed with a Mini-Protean II apparatus (Bio-Rad, Hercules, USA). Samples were thawed in an ice bath, briefly sonicated with UPS200S sonicator (Hielscher Ultrasonics, Teltow, Germany), mixed with 2× Laemmli sample buffer and dithiothreitol, and denatured by heating at 100 °C before loading. Page Ruler Plus pre-stained standard (Fermentas, Waltham, MA, USA) was used for molecular weight comparison. Proteins were transferred to nitrocellulose membrane (GE Healthcare Life Sciences, Marlborough, MA, USA) using wet transfer at 100 V for 90 min. Membranes were blocked in 5% non-fat dried milk in TBS with 0.05% Tween-20 (TBST; 50 mM Tris-HCl, 150 mM NaCl, 0.05% Tween 20, pH 7.5) and incubated overnight at 4 °C with mouse anti-FLAG IgG (Proteintech Group, Chicago, IL, USA; 1:1000) in 5% non-fat dried milk in TBST. Following three washes with TBST, membranes were incubated for 2 h with peroxidase conjugated secondary goat anti-mouse IgG (Jackson ImmunoResearch, West Grove, PA, USA; 1:5000) in 5% non-fat dried milk in TBST. After three further washes with TBST, membranes were incubated with Lumi-Light chemiluminescent reagent (Roche, Basel, Switzerland). Images were acquired using a ChemiDoc MP Imaging System (Bio-Rad, Hercules, CA, USA).

### 4.7. Flow Cytometry

For flow cytometry, 20 µL of a cell culture in the stationary phase was added to 500 µL of Tris-buffered saline (TBS; 50 mM Tris-HCl, 150 mM NaCl, pH 7.5) and centrifuged for 5 min at 5000× *g* and 4 °C. The pellet was resuspended in 200 µl of dialyzed Ni-NTA purified p19-trx (50 µg/mL in PBS buffer with carrier protein SuperBlock). After 2 h of incubation at RT with constant shaking at 100 rpm, cells were washed three times with 200 µL 0.1% TBS-Tween (TBST) and resuspended in 200 μL of TBS with mouse anti-human IL-23 p19 IgG (BioLegend, San Diego, CA, USA; diluted 1:500). After 2 h of incubation at RT with constant shaking at 100 rpm, cells were washed three times with 200 µL 0.1% TBS-Tween (TBST) and resuspended in 200 μL of TBS with Alexa Fluor 488-conjugated goat anti-mouse IgG (Invitrogen, Carlsbad, CA, USA; diluted 1:500). 2 h incubation at RT was repeated and cells were washed again three times with 200 μL TBST and finally resuspended in 500 µL TBS. Samples were analyzed with a FACSCalibur flow cytometer (Becton Dickinson, Franklin Lakes, NJ, USA) using excitation at 488 nm and emission at 530 nm in the FL1 channel. The results are presented as mean fluorescence intensity (MFI) values of at least 20,000 lactococcal cells. The result was expressed as the average of at least three independent experiments. Similar protocol was applied for the detection of FLAG-tagged ILP fusion proteins. Rabbit anti-FLAG IgG (Proteintech Group, Chicago, IL, USA; diluted 1:500) was used as primary antibody and Alexa Fluor 488-conjugated donkey anti-rabbit IgG (Invitrogen, Carlsbad, CA, USA; diluted 1:500) was used as a secondary antibody. All the experiments were performed in three biological replicates.

### 4.8. IL-23 Binding Assay 

The concentration of IL-23 was determined with Human IL-23 ELISA development kit (HRP) from Mabtech. Different numbers of *L. lactis* cells expressing ILP fusion proteins were centrifuged (5000× *g*, 5 min, 4 °C) and resuspended in 200 μL of incubation buffer (PBS with 0.05% Tween and 0.1% BSA) containing various concentrations of IL-23 standard and incubated for 2 h at room temperature (RT) with gentle shaking. Cells were then removed by centrifugation (5000× *g*, 5 min, 4 °C) and the concentrations of the remaining cytokines in the supernatant were determined according to the ELISA manufacturer’s instructions. Briefly, Nunc Maxisorp 96-well plates were coated with recommended concentrations of IL-23-binding antibodies overnight at 4 °C. 100 μL of samples were then added and incubated for 2 h at RT. Wells were washed five times with 200 μL of PBS containing 0.05% Tween (PBST). 100 μL per well of recommended concentration of biotinylated monoclonal antibodies against IL-23 were added and incubated at RT for 1 h. Wells were washed again for five times with 200 μL of PBST. 100 μL of streptavidin-HRP diluted 1:1000 was added to the plate and incubated for 1 h at RT. The plate was washed again for five times with PBST and 100 μL of 3,3′,5,5′-tetramethylbenzidine (TMB) substrate (Sigma-Aldrich, St. Louis, MO, USA) was added. The reaction was stopped after 15 min by the addition of 50 μL of 2 M sulfuric acid. Absorbances were read at 450 nm using an Infinite M1000 microplate-reader (Tecan, Mannedorf, Switzerland).

### 4.9. Statistics

Data were analyzed with GraphPad Prism 5.0 software. Mean fluorescence intensitites (MFIs), obtained by flow cytometry, were compared by ANOVA followed by post-hoc Bonferroni multiple pair comparison. Relevant pairs with significant differences (*p* < 0.05) were highlighted. Concentrations of IL-23, obtained by ELISA, were compared to control using *t*-test. Significant differences (*p* < 0.05) were highlighted.

### 4.10. Modeling of ILP-p19 and Interactions

The homology model of the mouse p19 was prepared using the MODELLER 9v14 suite of programs [43] based on the homologous structure of human p19 (PDB ID 3duh [44], protein sequence identity 71.9% and the similarity 90.4%), where the missing residues from the p19 loop regions were added using the loopmodel function of MODELLER. The homology model was relaxed using an implicit solvation parm96 molecular dynamics simulation. The MD parameters were assigned in Zephyr graphical interface [45] and 20 ns simulation was performed using GPU accelerated version of gromacs package [46]. The structure of studied ABD variants (ILP030, ILP317, and ILP323) was modeled based on the ABDwt structure (PDB ID 1gjt [47]). All the necessary sequence alignments were performed employing Muscle program [48]. Resulting three-dimensional structures were subjected to the flexible side chain docking performed using a local copy of the ClusPro server [49,50].

## 5. Conclusions

Collectively, this work contributes to development of new types of recombinant bacteria of food origin capable of displaying and secreting engineered binding proteins as non-immunoglobulin-blocking alternatives. The generated and characterized recombinant bacteria strains represent novel tools for in vivo testing of IL-23 anti-inflammatory agents that might be useful for treatment of intestinal inflammation.

## Figures and Tables

**Table 1:** Bacterial strains, plasmids and primers used in the study.

Strain, Plasmid, or Gene	Relevant Features or Sequence (5′–3′)	Reference or Source
**Strain**		
*E. coli*		
DH5α	endA1 glnV44 thi-1 recA1 relA1 gyrA96 deoR F- Φ80d*lacZ*ΔM15 Δ(*lacZYA-argF*)U169, hsdR17(rK- mK+), λ–	Invitrogen
TOP10	F– mcrA Δ(mrr-hsdRMS-mcrBC) Φ80lacZΔM15 ΔlacX74 recA1 araD139 Δ(ara leu) 7697 galU galK rpsL (StrR) endA1 nupG	Life technologies
BL21 λ(D3)	*E. coli* B F – dcm ompT hsdS (rB– mB–) gal λ(DE3)	[41]
*L. lactis*		
NZ9000	MG1363 *nisRK* Δ*pepN*	[42]
**Plasmid**		
pNZ8148	pSH71 derivative, P*nisA*, CmR, nisin-controlled expression	[42]
pSDLBA3b	pNZ8148 containing gene fusion of Usp45 signal peptide, B domain and cA	[32]
pET-ILP030	pET28b containing a fusion gene of ILP030, tolA protein and AviTag consensus	[14]
pET-ILP317	pET28b containing a fusion gene of ILP317, tolA protein and AviTag consensus	[14]
pET-ILP323	pET28b containing a fusion gene of ILP323, tolA protein and AviTag consensus	[14]
pSD-ILP030	pNZ8148 containing gene fusion of Usp45 signal peptide, ILP030 and cA	This work
pSD-ILP317	pNZ8148 containing gene fusion of Usp45 signal peptide, ILP317 and cA	This work
pSD-ILP323	pNZ8148 containing gene fusion of Usp45 signal peptide, ILP323 and cA	This work
pSD-ADN23	pNZ8148 containing gene fusion of Usp45 signal peptide, ADN23 and cA	[38]
pSD-ILP030-FLAG	pNZ8148 containing gene fusion of Usp45 signal peptide, ILP030 and cA	This work
pSD-ILP317-FLAG	pNZ8148 containing gene fusion of Usp45 signal peptide, ILP317 and cA	This work
pSD-ILP323-FLAG	pNZ8148 containing gene fusion of Usp45 signal peptide, ILP323 and cA	This work
pSD-ADN23-FLAG	pNZ8148 containing gene fusion of Usp45 signal peptide, ADN23 and cA	This work
pET-DH-TRX-p19	pET28b containing a fusion gene of double-His-tag, Thioredoxin and p19 protein	This work
**Primer**		
ILP030-F	TGGATCCTTAGCTGAAGCTAAAGTC	This work
ILP030-R	AGAATTCAGGTAAATTAGCTAAAATACG	This work
ILP317-R	AGAATTCAGGTAAAGGAGCTAAAATACTATC	This work
ILP323-R	AGAATTCAGGTAAACGAGCTAAAATAACATC	This work
Usp1-NcoI	ATAACCATGGCTAAAAAAAAGATTATCTCAGCTATTTTAATG	[32]
FLAG_Bam_R	GGATCCTTTATCATCGTCGTCTTTATAATCAGCGTAAACACCTGACAACG	This work
19-F-NheI	GGGCTAGCTAGCAGAGCTGTGCCTGGGGGC	This work
p19-R-XhoI	GCGCCTCGAGGGGACTCAGGGTTGCTGCTC	This work
